# Severe acute respiratory syndrome coronavirus (SARS-CoV)-2 infection induces dysregulation of immunity: *in silico* gene expression analysis

**DOI:** 10.7150/ijms.52256

**Published:** 2021-01-01

**Authors:** Yen-Hung Wu, I-Jeng Yeh, Nam Nhut Phan, Meng-Chi Yen, Hsin-Liang Liu, Chih-Yang Wang, Hui-Ping Hsu

**Affiliations:** 1 Department of Emergency Medicine, Kaohsiung Medical University Hospital, Kaohsiung Medical University, Kaohsiung 80708, Taiwan.; 2 Graduate Institute of Clinical Medicine, College of Medicine, Kaohsiung Medical University, Kaohsiung 80708, Taiwan.; 3 NTT Institute of Hi-Technology, Nguyen Tat Thanh University, Ho Chi Minh City 700000, Vietnam.; 4 PhD Program for Cancer Molecular Biology and Drug Discovery, College of Medical Science and Technology, Taipei Medical University, Taipei 11031, Taiwan.; 5 Graduate Institute of Cancer Biology and Drug Discovery, College of Medical Science and Technology, Taipei Medical University, Taipei 11031, Taiwan.; 6 Department of Surgery, National Cheng Kung University Hospital, College of Medicine, National Cheng Kung University, Tainan 70101, Taiwan.; 7 Department of Biostatistics, Vanderbilt University Medical Center, Nashville, TN 37232, USA.

**Keywords:** coronavirus, SARS-CoV-2, COVID-19, interferon, immune system

## Abstract

Highly pathogenic coronaviruses (CoVs) induce acute respiratory distress syndrome, and the severe acute respiratory syndrome coronavirus (SARS-CoV)-2 has caused a pandemic since late 2019. The diversity of clinical manifestations after SARS-CoV-2 infection results in great challenges to diagnose CoV disease 2019 (COVID-19). There is a growing body of published research on this topic; however, effective medications are still undergoing a long process of being assessed. In the search for potential genetic targets for this infection, we applied a holistic bioinformatics approach to study alterations of gene signatures between SARS-CoV-2-infected cells and mock-infected controls. Two different kinds of lung epithelial cells, A549 with angiotensin-converting enzyme 2 (ACE2) overexpression and normal human bronchial epithelial (NHBE) cells, were infected with SARS-CoV-2. We performed bioinformatics analyses of RNA-sequencing in this study. Through a Venn diagram, Database for Annotation, Visualization and Integrated Discovery, Gene Ontology, Ingenuity Pathway Analysis, and Gene Set Enrichment Analysis, the pathways and networks were constructed from commonly upregulated genes in SARS-CoV-2-infected lung epithelial cells. Genes associated with immune-related pathways, responses of host cells after intracellular infection, steroid hormone biosynthesis, receptor signaling, and the complement system were enriched. Dysregulation of the immune system and malfunction of interferon contribute to a failure to kill SARS-CoV-2 and exacerbate respiratory distress in severely ill patients. Current findings from this study provide a comprehensive investigation of SARS-CoV-2 infection using high-throughput technology.

## Introduction

Coronaviruses (CoVs) are RNA viruses that cause diseases related to the respiratory system in both humans and animals 1. Currently, several CoV strains have been reported, including HCoV-OC43, HCoV-229E, HCoV-NL63, and HCoV-HKU1, which infect humans and induce respiratory tract infections 2. These CoVs can cause less-pathogenic endemic diseases in infected hosts. However, in 2003, a severe pandemic occurred resulting in more than 8422 cases and about 916 related deaths caused by another strain of CoV, namely severe acute respiratory syndrome (SARS)-CoV 3-6. In 2012, another CoV strain, namely the Middle East respiratory syndrome (MERS)-CoV, that infected humans, bats, and camels also caused more than 4494 cases and 858 deaths 7. Most recently, in 2019, a new strain of CoV, the so-called 2019-novel coronavirus (2019-nCoV/SARS-CoV-2), is causing a global pandemic affecting almost every country, causing unaccountable losses from taking human lives to damaging the world's economy. As of Dec 18, 2020, according to statistical reports from the World Health Organization (WHO) (https://www.who.int/emergencies/diseases/novel-coronavirus-2019), over 73,996,237 laboratory-confirmed cases and 1,663,474 deaths worldwide were caused by SARS-CoV-2. This CoVs outbreak again corroborates that CoVs can definitely cause severe disease in humans once infected. Investigating suitable treatment approaches is peremptory; however, there are no clear or widely recognized standard methods for dealing with this infection currently.

According to previous research, A549 lung adenocarcinoma cells are not vulnerable to SARS-CoV or SARS-CoV-2 infection 8, 9. A549 cells express dipeptidyl peptidase 4, which is the receptor for MERS-CoV 10. A549 cells are human adenocarcinomatous alveolar type II cells, and they turned out to be susceptible to SARS-CoV-2 after overexpressing angiotensin-converting enzyme 2 (ACE2) receptors 8. Normal human bronchial epithelial (NHBE) cells are normal epithelial cells that are commonly used as *in vitro* lung models 11, 12. ACE2 receptors are an entry point for CoVs, and ACE2 expression was detected in NHBE cells 13. These cells are highly beneficial for studying CoV infections and developing therapeutic strategies in experimental subjects.

Over the last decade, high-throughput technologies have emerged as major players in producing huge amounts of output data for multipurpose research from genomics to proteomics. These technologies allow researchers to rapidly acquire hundreds to several thousand gene expression profiles in each experiment 14-17. Leveraging these tools and publicly available datasets for SARS-CoV-2, we attempted to use a high-throughput approach to search for predictive markers and therapeutic strategies for CoVs. We integrated multiple bioinformatics tools for whole-profile gene expressions of SARS-CoV-2-infected human lung epithelial cells. We analyzed RNA sequencing (RNA-Seq) data and explored genetic signatures associated with SARS-CoV-2-infected ACE2-expressed-A549 and NHBE cells. In addition, downstream regulatory pathways were predicted. The candidate genes were evaluated as the potentially therapeutic targets for SARS-CoV-2 infection. The present study can provide essential evidence of regulatory networks in SARS-CoV-2-infected disease.

## Materials and Methods

### Bioinformatics and high-throughput database analyses

We acquired RNA-Seq data of ACE2-expressed-A549 and NHBE cells with SARS-CoV-2 infection from the National Center for Biotechnology Information Gene Expression Omnibus (NCBI GEO), with accession number GSE147507 18 and analyzed those data with the CLC Genomics Workbench (https://digitalinsights.qiagen.com). To acquire gene symbols, gene IDs were mapped to Ensembl features using the biomaRt package vers. 2.26.1 and Gene Ontology (GO)-Elite platforms. The clustering of genes was based on their messenger (m)RNA expression profiles using pheatmap vers. 1.0.12 of R software 19-23. Signals were processed and normalized as we previously described 24-32. The Database for Annotation, Visualization, and Integrated Discovery (DAVID, vers. 6.8) was used for clustering analyses of genes of interest. This clustering algorithm uses biological functions, signaling pathways, and associated diseases.

In order to study acute infection of SARS-CoV-2, ACE2-expressed-A549 and NHBE cells were infected with SARS-CoV-2 and mock controls for 24 h. Differential expressions of genes were sequenced on the Illumina NextSeq 500 system. We set infected cells versus mock controls to >2.0 for both ACE2-expressed-A549 and NHBE cells as cutoff points. Differentially expressed genes (DEGs) were calculated, and *P* values of <0.05 were considered significant. The list of significantly different genes was imported to the gene ontology (GO) database to construct biological processes and associated diseases 33. To construct the biological regulatory networks of targeted genes, Gene Set Enrichment Analysis (GSEA) software was used for enrichment 34. A *P* value of <0.05 was selected as the cutoff point for the enrichment analysis.

### Pathway and network enrichment analyses

To further investigate the signal pathways related to SARS-CoV-2-infected cells, an Ingenuity pathway analysis (IPA) was used with data input as the list of DEGs with significant differences from SARS-CoV-2-infected human lung epithelial cells. The *P* value (Benjamini-Hochberg) of <0.05 was selected as the cutoff for statistically significant differences.

## Results

### GSEA of SARS-CoV-2-infected human lung epithelial cells

**Figure 1:**
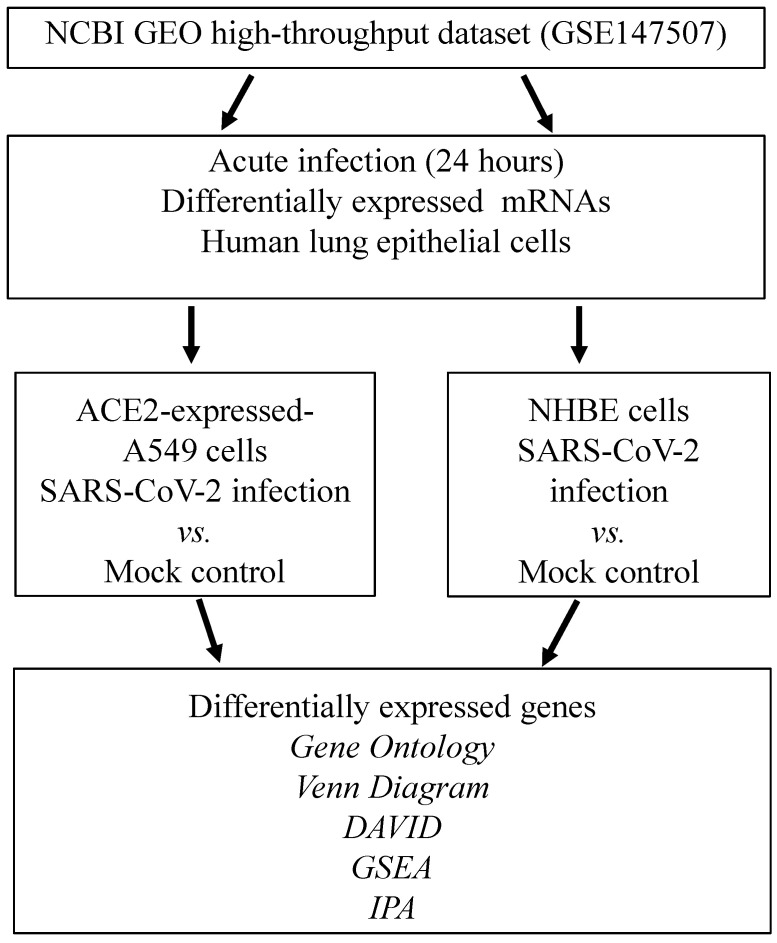
** Schematic workflow illustrating the study design.** Severe acute respiratory syndrome coronavirus (SARS-CoV)-2-infected ACE2-expressed-A549 and normal human bronchial epithelial (NHBE) cells were acquired from the GSE147507 dataset in the National Center for Biotechnology Information Gene Expression Omnibus (NCBI GEO) databases. Highly expressed genes were merged in a Venn diagram analysis. Shared genes from the two datasets were analyzed by the DAVID, GO, IPA, and GSEA. Abbreviation: ACE2, angiotensin-converting enzyme 2; DAVID, Database for Annotation, Visualization, and Integrated Discovery; GO, gene ontology; GSEA, Gene Set Enrichment Analysis; IPA, ingenuity pathway analysis; NHBE cells, normal human bronchial epithelial cells; SARS-CoV-2, severe acute respiratory syndrome coronavirus-2.

We attempted to identify DEGs in SARS-CoV-2-infected cells compared to mock-infected controls. Experiments were conducted on ACE2-expressed-A549 and NHBE cells. Statistically significantly upregulated genes were analyzed with numerous bioinformatics tools, including a Venn diagram for the intersection of two studies, DAVID for associated functions, GO Elite for biological processes, IPA for regulated networks, and GSEA for biological regulation (Figure 1).

### GO analysis of SARS-CoV-2-infected human lung epithelial cells

**Figure 2:**
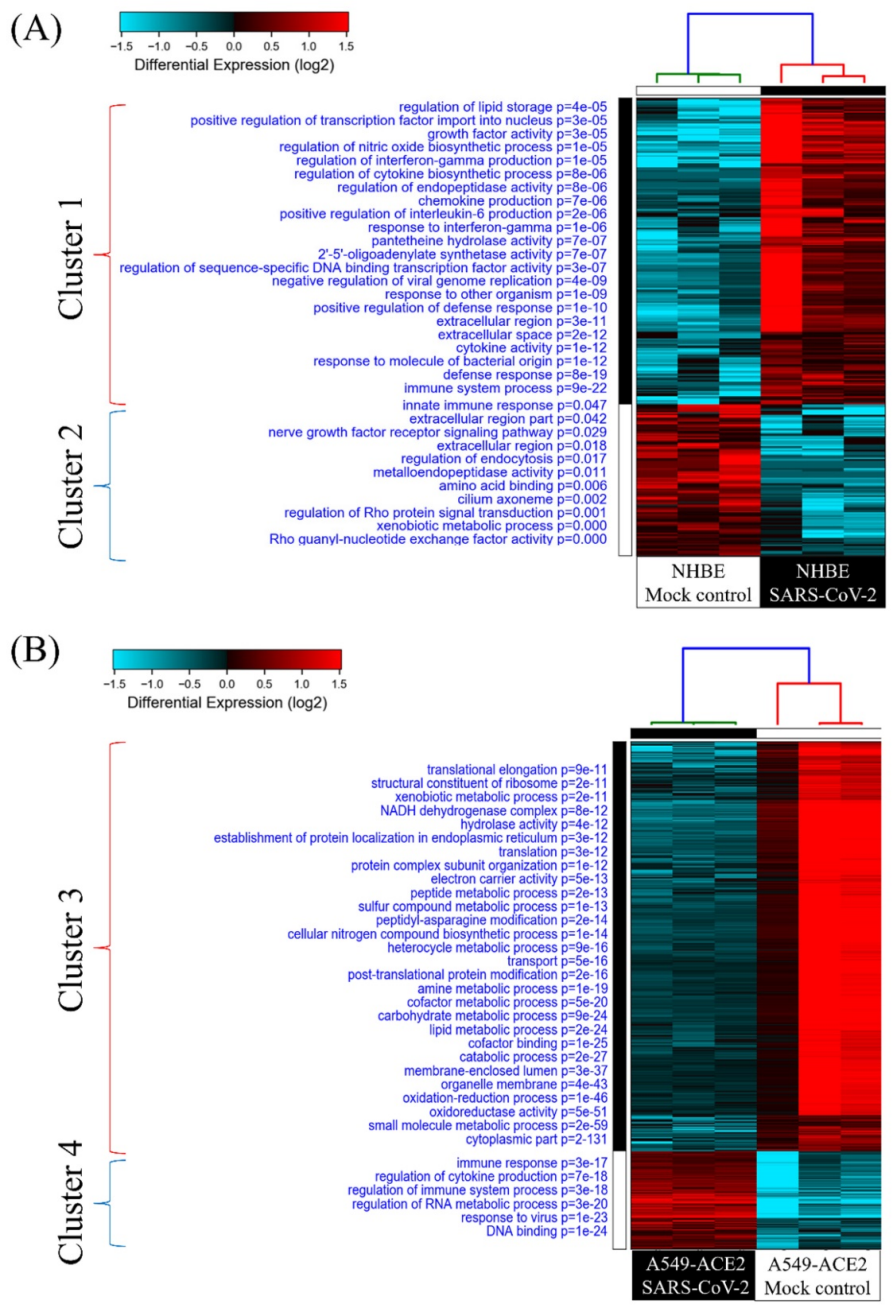
** Heatmap visualization of enriched gene ontology (GO) from severe acute respiratory syndrome coronavirus (SARS-CoV)-2-infected lung epithelial cells compared to mock-infected control group.** (A) Comparison between SARS-CoV-2-infected and mock-infected normal human bronchial epithelial (NHBE) cells. Upregulated genes were analyzed by a GO enrichment analysis for associated pathways. Cluster 1 pathways were derived from upregulated genes in SARS-CoV-2-infected cells, and cluster 2 pathways were of downregulated genes. (B) Comparison between SARS-CoV-2-infected and mock-infected A549 cells transduced with a vector expressing human ACE2. Upregulated genes were analyzed by a GO enrichment analysis for associated pathways. Cluster 3 pathways were derived from downregulated genes in SARS-CoV-2-infected cells and cluster 4 from upregulated genes.

To investigate cellular responses of SARS-CoV-2-infected lung epithelial cells, we collected gene expression values from the GSE147507 dataset. Comparing SARS-CoV-2-infected NHBE cells and mock-infected controls showed two clusters of genes. Genes in group 1 were upregulated in SARS-CoV-2-infected NHBE cells, and the associated pathways were enriched according to the GO analysis (Figure 2A). The most significant pathways of upregulated genes were "immune system process (*P* = 9 × 10^-22^)", "defense response (*P* = 8 × 10^-19^)", "response to molecules of bacterial origin (*P* = 1 × 10^-12^)", and "cytokine activity (*P* = 1 × 10^-12^)”. Cellular responses of SARS-CoV-2-infected A549 cells transduced with a vector expressing human ACE2 were analyzed with the same method, and enriched pathways are shown in Figure 2B. Data revealed that the most important pathways of uprelated genes were "DNA binding (*P* = 1 × 10^-24^)", "response to virus (*P* = 1 × 10^-23^)", "regulation of RNA metabolic process (*P* = 3× 10^-20^)", and " regulation of immune system process (*P* = 3 × 10^-18^)". These results are consistent with recent COVID-19 research, which demonstrated an association of SARS-CoV-2 infection with immune response in patients 35. Furthermore, in the progression and development of this infectious disease, declines in circulating natural killer cell levels were also linked to the disease severity 36.

### Mutual gene signatures in ACE2-expressed-A549 and NHBE cells after SARS-CoV-2 infection

**Figure 3:**
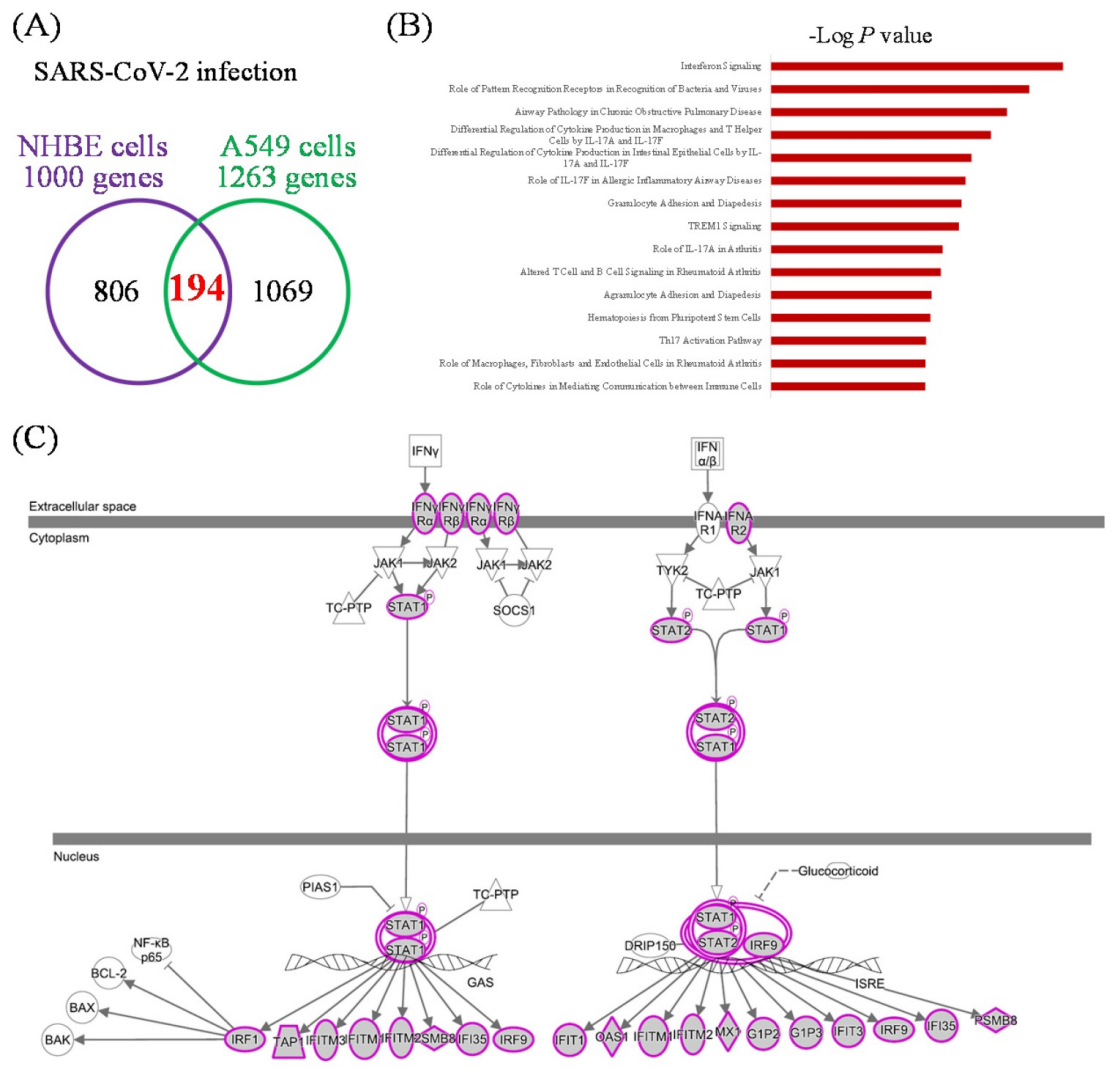
** Common expressed genes, pathways, and networks from severe acute respiratory syndrome coronavirus (SARS-CoV)-2-infected lung epithelial cells.** (A) Differentially highly expressed genes from two kinds of lung epithelial cells with SARS-CoV-2 infection were merged in a Venn diagram. (B) Ingenuity Pathway Analysis (IPA) software was used to analyze shared upregulated genes of ACE2-expressed-A549 and normal human bronchial epithelial (NHBE) cells. SARS-CoV-2-associated pathways and networks are listed with the negative logarithmic form of the *P* value. (C) Detailed pathway map of "interferon signaling".

There were 1000 upregulated genes in SARS-CoV-2-infected NHBE cells compared to mock-infected controls, and 1263 genes in SARS-CoV-2-infected ACE2-expressed-A549 cells compared to the controls (Figure 3A). There were 194 genes in common. We imported these 194 genes into the IPA platform to explore characteristic signatures of gene expressions in these two lung epithelial cell lines. The highest enriched common pathways were "interferon (IFN) signaling (*P* = 1.58 × 10^-11^)", "role of pattern recognition receptors in recognition of bacteria and viruses (*P* = 2.82 × 10^-10^)", and "airway pathology in chronic obstructive pulmonary disease (*P* = 1.86 × 10^-9^)" (Figure 3B, Table 1). Details of IFN signaling are shown in Figure 3C, such as extracellular IFN-α/β interacting with IFN-α receptor 1/2 (IFNAR1/2), and extracellular IFN-γ being associated with IFN-γRα/β. Activation of intracellular signal transducer and activator of transcription (STAT) signaling initiates transcription of downstream genes.

### GSEA of SARS-CoV-2-infected cells

**Figure 4:**
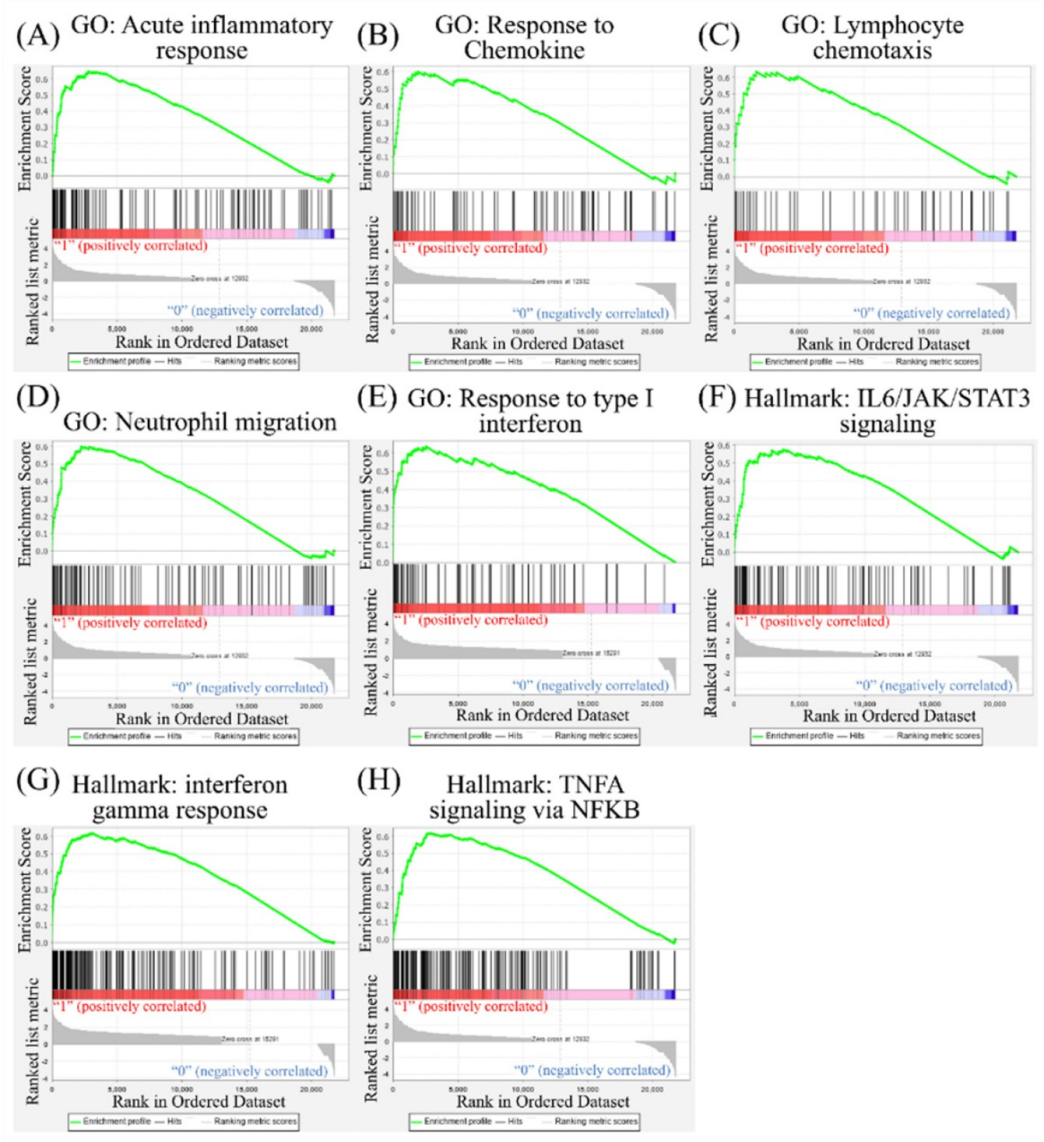
** Gene Set Enrichment Analysis (GSEA) showing enriched immune-related pathways in severe acute respiratory syndrome coronavirus (SARS-CoV)-2 infected cells.** The Gene Ontology (GO) and Hallmark platforms were used to analyze upregulated genes from SARS-CoV-2-infected ACE2-expressed-A549 and normal human bronchial epithelial (NHBE) cells relative to mock-infected controls. An enrichment score threshold of >0 was set for upregulation. In the middle portion of each figure, members of each gene set are represented as a single bar. For positive correlations highlighted in red, bars shown before the peak score contributed the most to that score, whereas for negative correlations highlighted in blue, bars shown right after the peak score contributed the most to that score. In the last portion of each graph, the ranking metric shows the correlation of the gene set with either the first or second phenotype. (A) Acute inflammatory response. (B) Response to chemokines. (C) Lymphocyte chemotaxis. (D) Neutrophil migration. (E) Response to type I interferon. (F) Interleukin (IL)-6/Janus kinase (JAK)/signal transducer and activator of transcription 3 (STAT3) signaling. (G) Interferon-gamma response. (H) Tumor necrosis factor-α (TNFA) signaling via nuclear factor-κB (NFKB).

**Figure 5:**
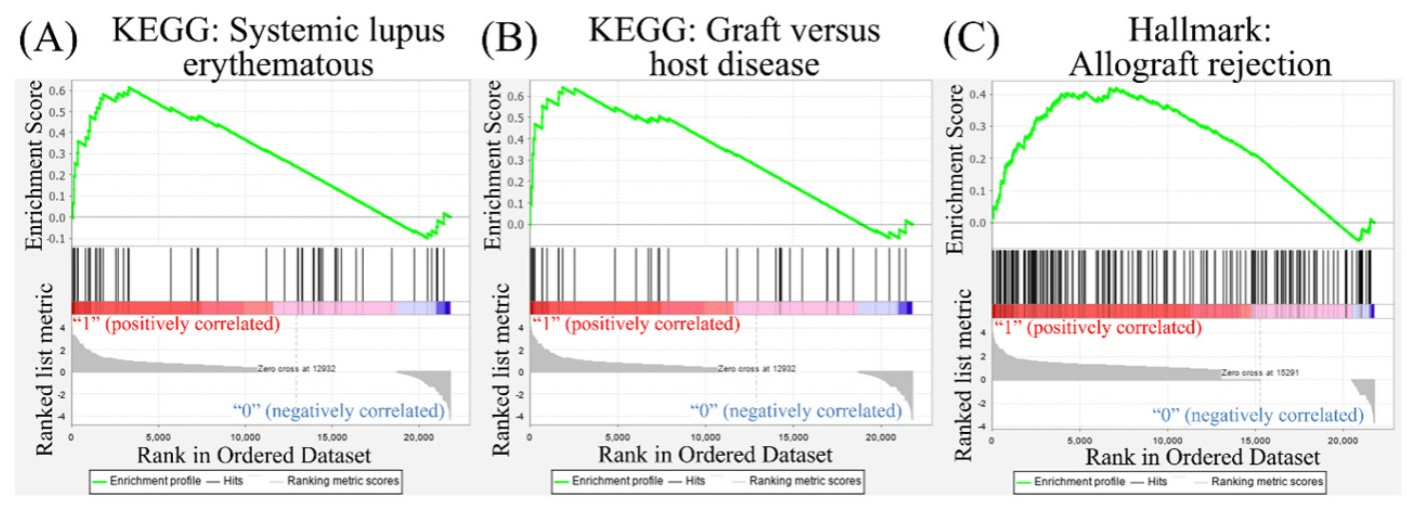
** Disease associated with dysregulation of the immune system in a Gene Set Enrichment Analysis (GSEA).** The Kyoto Encyclopedia of Genes and Genomes (KEGG) and Hallmark platforms were utilized to analyze upregulated genes from severe acute respiratory syndrome coronavirus (SARS-CoV)-2-infected ACE2-expressed-A549 and normal human bronchial epithelial (NHBE) cells. SARS-CoV-2-infected cells were compared to mock-infected controls. (A) Systemic lupus erythematosus. (B) Graft versus host disease. (C) Allograft rejection.

**Figure 6:**
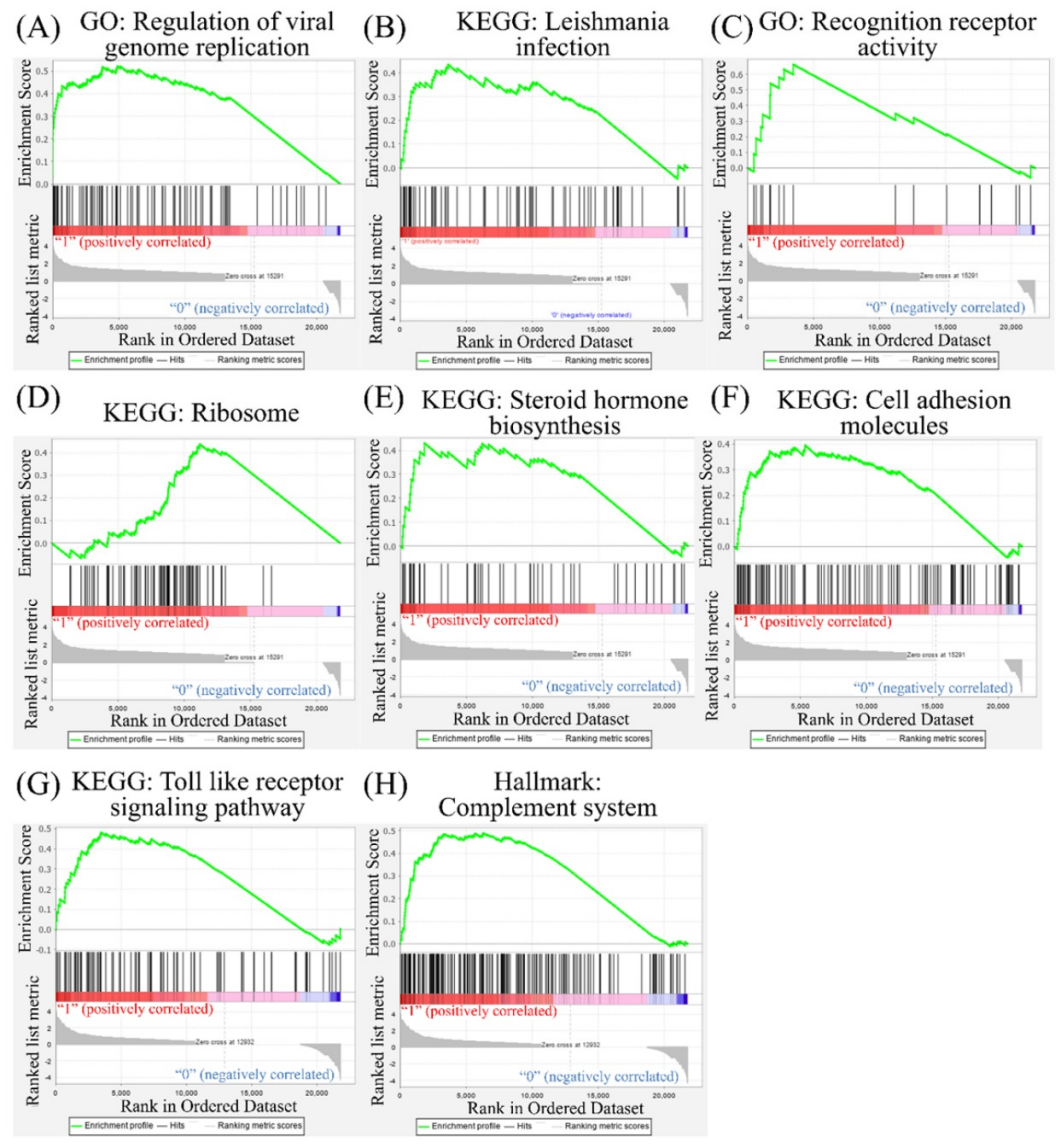
** Other pathways in the Gene Set Enrichment Analysis (GSEA).** The Kyoto Encyclopedia of Genes and Genomes (KEGG), Gene Ontology (GO), and Hallmark platforms were utilized to analyze upregulated genes from severe acute respiratory syndrome coronavirus (SARS-CoV)-2-infected ACE2-expressed-A549 and normal human bronchial epithelial (NHBE) cells compared to mock-infected controls. (A) Regulation of viral genome replication. (B) *Leishmania* infection. (C) Recognition of receptor activity. (D) Ribosomes. (E) Steroid hormone biosynthesis. (F) Cell adhesion molecules. (G) Toll-like receptor signaling pathway. (H) Complement system

Leveraging public databases such as Hallmark and KEGG, we verified the importance of the enriched pathways from the shared 194 upregulated genes between SARS-CoV-2 infected A549 and SARS-CoV-2-infected NHBE cells relative to mock-infected controls (Figure 2). Several immune-related networks were enriched, including acute inflammation, chemokine, chemotaxis, neutrophil migration, response to IFN, and interleukin (IL)-6-related signaling pathways (Figure 4). Some diseases associated with dysregulation of the immune system were also positively correlated, including systemic lupus erythematosus, graft versus host disease, and allograft rejection (Figure 5). Host responses after SARS-CoV-2 infection were also enriched, e.g., regulation of viral genome replication, *Leishmania* infection, recognition receptor activity, steroid hormone biosynthesis, cell adhesion molecules, toll-like receptor (TLR) signaling pathway, and the complement system (Figure 6).

## Discussion

The COVID-19 pandemic has induced a global health crisis with tremendous impacts on humans. Many researchers have focused on this disease; however, there are currently still numerous unsolved problems. In the present study, we used two cell models from different human lung epithelial cell lines (ACE2-expressed-A549 and NHBE cells) and used them to compare SARS-CoV-2-infected and mock-infected cells. We concentrated on a 24-h model study of cellular responses after SARS-CoV-2 infection. Our results showed activation of immune-related networks, especially IFN signaling. Immune cell migration and chemotaxis were also upregulated. Increased expression of disease-associated genes were detected, including those correlated with immune-dysregulated diseases. The current data provide a comprehensive understanding of acute SARS-CoV-2 infection, and dysregulation of the immune system might be a future treatment target.

CoVs were reported to be correlated with cytokine storms and inflammation in previous studies 37, 38. Serum levels of IL family members are elevated in patients experiencing a cytokine storm, including IL-6, IL-1, IL-1β, tumor necrosis factor (TNF), and C-C chemokine ligand 2 (CCL2) 39, 40. Targeting these cytokines should control complications due to the cytokine storm; however, results of a clinical trial were equivocal 37. Other cytokines may also play roles in SARS-CoV-2 infection. The accumulation of transforming growth factor (TGF)-β in the lungs induces dysregulation of coagulation and fibrinolytic pathways, resulting in lung fibrosis 41. Expressions of IFN regulatory factor (IRF)-1, IL-6, IL-8, and IL-18 in lung tissues were elevated in acute respiratory distress syndrome 42, 43. SARS-CoV-2 relies on ACE2 and transmembrane serine protease 2 (TMPRSS2) to enter cells 44.

ACE2 is a human IFN-stimulated gene in lung type II pneumocytes 45. The immune response with type I IFN (IFN-α/β) is one kind of innate immunity against the virus. However, CoVs are capable of interrupting IFN responses by viral proteins 46, 47. In our study, we used GSEA, GO, KEGG, and Hallmark platforms, and revealed that responses to IFN-α/γ were upregulated in SARS-CoV-2-infected ACE2-expressed-A549 cells (Figure 2). By merging upregulated genes in NHBE and ACE2-expressed-A549 cells, IFN signaling was found to be the most crucial pathway (Figure 3). In other platforms, a positive correlation between responses to type I IFN or IFN-γ or IL-6 and SARS-CoV-2 infection were detected with other cytokines (Figure 4). Gene signatures of acute SARS-CoV-2-infected cells were similar to those of diseases induced by dysregulation of the immune system (Figure 5). Dysregulated immune system may restrain the killing ability of IFN signaling and SASRS-CoV-2 survive in these conditions. Modulating the host response to stable homeostasis is an important part of treating SARS-CoV-2 infection.

According to a previous study, the androgen receptor is required for transcription of the *TMPRSS2* gene 48, 49, which is the protease for spike proteins on CoVs 50. In the present study, positive correlations of steroid hormone biosynthesis with SARS-CoV-2 infection were detected (Figure 6). We also recognized responses of host cells after SARS-CoV-2 infection, including regulation of viral genome replication, *Leishmania* infection, and recognition receptor activity. The protozoan *Leishmania* is a genus of parasites, and *Leishmania* infection induces high expression of IL-17 51. Besides, recognition receptor activity is the cellular response after binding to specific receptors. Binding of SARS-CoV-2 to membranous ACE2 receptors of host cells initiates specific downstream actions that support the survival of CoVs 29. Meanwhile, upregulation of cell adhesion molecules, TLR signaling pathways, and the complement system was also discovered. Binding of SARS-CoV-2 with TLRs induces the release of pro-inflammatory cytokines, including IL-1β and IL-6 52, 53. The complement system is one of the essential components of innate immunity after viral infection. Activation of the complement system contributes to a dysregulated inflammatory response, and recent reports presumed the complement system to be a target for treating severe illness due to COVID-19 54, 55. Our bioinformatics analysis supported most of that research. The cellular response is complicated after SARS-CoV-2 infection, and a comprehensive understanding of these networks will help us find effective therapeutic agents.

Collectively, the present study focused on the response of lung epithelial cells after SARS-CoV-2 infection. Dysregulation of the immune system, production of steroid hormones, alteration of cell adhesion molecules, and a maladaptive complement system all contribute to clinical sequelae of lung fibrosis and respiratory distress. The current findings from our study could contribute to the battle against SARS-CoV-2 using high-throughput methods, which could shorten the time consumed for target screening and provide an underlying mechanism via a network enrichment analysis. These pathways and networks could provide potential targets for prospective experimental studies related to SARS-CoV-2 treatment.

## Figures and Tables

**Table 1:** Ingenuity Pathway Analysis (IPA) of potential interactions among upregulated genes of ACE2-expressed-A549 and normal human bronchial epithelial (NHBE) cells infected for 24 h with severe acute respiratory syndrome coronavirus (SARS-CoV)-2 compared to a control group in the GSE147507 dataset.

Canonical Pathways	*P* value	Molecules
Interferon Signaling	1.58E-11	IFI35,IFI6,IFIT1,IFIT3,IFITM1,IFITM3,ISG15,MX1,OAS1
Role of Pattern Recognition Receptors in Recognition of Bacteria and Viruses	2.82E-10	CSF2,IL1A,IL6,IRF7,LIF,LTB,NFKB2,NOD2,OAS1,OAS2,OAS3,PTX3,TNF
Airway Pathology in Chronic Obstructive Pulmonary Disease	1.86E-09	CCL2,CCL20,CSF2,CXCL1,CXCL3,IL1A,IL23A,IL6,LIF,LTB,TNF
Differential Regulation of Cytokine Production in Macrophages and T Helper Cells by IL-17A and IL-17F	7.24E-09	CCL2,CSF2,CSF3,CXCL1,IL6,TNF
Differential Regulation of Cytokine Production in Intestinal Epithelial Cells by IL-17A and IL-17F	3.80E-08	CCL2,CSF2,CSF3,CXCL1,IL1A,TNF
Role of IL-17F in Allergic Inflammatory Airway Diseases	6.31E-08	CCL2,CSF2,CXCL1,CXCL10,IL6,MMP13,NFKB2
Granulocyte Adhesion and Diapedesis	8.91E-08	CCL2,CCL20,CSF3,CXCL1,CXCL10,CXCL11,CXCL2,CXCL3,IL1A,MMP13,TNF
TREM1 Signaling	1.12E-07	CCL2,CSF2,CXCL3,IL6,NFKB2,NOD2,STAT5A,TNF
Role of IL-17A in Arthritis	4.47E-07	CCL2,CCL20,CXCL1,CXCL3,MMP13,NFKB2,NFKBIA
Altered T Cell and B Cell Signaling in Rheumatoid Arthritis	5.13E-07	CSF2,IL1A,IL23A,IL6,LTB,NFKB2,RELB,TNF
Agranulocyte Adhesion and Diapedesis	1.15E-06	CCL2,CCL20,CXCL1,CXCL10,CXCL11,CXCL2,CXCL3,IL1A,MMP13,TNF
Hematopoiesis from Pluripotent Stem Cells	1.26E-06	CSF2,CSF3,IL1A,IL6,IL7,LIF
Th17 Activation Pathway	1.82E-06	CCL20,CSF2,IL23A,IL6,IRAK2,NFKB2,SOCS3
Role of Macrophages, Fibroblasts and Endothelial Cells in Rheumatoid Arthritis	1.91E-06	CCL2,CSF2,FRZB,IL1A,IL6,IL7,IRAK2,LTB,MMP13,NFKBIA,SOCS3,TNF,WNT3A
Role of Cytokines in Mediating Communication between Immune Cells	1.95E-06	CSF2,CSF3,IL1A,IL23A,IL6,TNF
IL-23 Signaling Pathway	2.29E-06	CSF2,IL23A,NFKB2,NFKBIA,SOCS3,TNF
IL-17A Signaling in Gastric Cells	2.29E-06	CCL20,CXCL1,CXCL10,CXCL11,TNF
Hepatic Cholestasis	3.24E-06	CSF2,CYP27A1,IL1A,IL6,IRAK2,LIF,LTB,NFKB2,NFKBIA,TNF
TNFR2 Signaling	5.01E-06	BIRC3,NFKB2,NFKBIA,TNF,TNFAIP3
Activation of IRF by Cytosolic Pattern Recognition Receptors	7.76E-06	IL6,IRF7,ISG15,NFKB2,NFKBIA,TNF
Role of Hypercytokinemia/hyperchemokinemia in the Pathogenesis of Influenza	8.32E-06	CCL2,CXCL10,IL1A,IL6,TNF
HMGB1 Signaling	8.51E-06	CCL2,CSF2,IL1A,IL6,LIF,LTB,NFKB2,RND1,TNF
IL-9 Signaling	9.55E-06	BCL3,NFKB2,SOCS3,STAT5A,TNF
IL-17A Signaling in Fibroblasts	1.29E-05	CCL2,IL6,NFKB2,NFKBIA,NFKBIZ
Dendritic Cell Maturation	1.55E-05	CSF2,IL1A,IL23A,IL6,LTB,NFKB2,NFKBIA,RELB,TNF
Role of Osteoblasts, Osteoclasts and Chondrocytes in Rheumatoid Arthritis	1.78E-05	BIRC3,CSF2,FRZB,IL1A,IL6,IL7,MMP13,NFKBIA,TNF,WNT3A
IL-17A Signaling in Airway Cells	1.91E-05	CCL20,CXCL1,CXCL3,IL6,NFKB2,NFKBIA
IL-10 Signaling	2.69E-05	IL1A,IL6,NFKB2,NFKBIA,SOCS3,TNF
Neuroinflammation Signaling Pathway	3.63E-05	BIRC3,CCL2,CXCL10,GABRR2,IL6,IRAK2,IRF7,NCF1,NFKB2,PLA2G4E,TNF
Toll-like Receptor Signaling	4.47E-05	IL1A,IRAK2,NFKB2,NFKBIA,TNF,TNFAIP3
Atherosclerosis Signaling	5.62E-05	CCL2,IL1A,IL6,MMP13,NFKB2,PLA2G4E,TNF
TNFR1 Signaling	6.31E-05	BIRC3,NFKB2,NFKBIA,TNF,TNFAIP3
Systemic Lupus Erythematosus In B Cell Signaling Pathway	8.51E-05	CSF2,IFIT3,IL1A,IL6,IRF7,ISG15,LIF,LTB,NFKB2,TNF
Lymphotoxin Receptor Signaling	1.00E-04	CXCL1,LTB,NFKB2,NFKBIA,RELB
Role of IL-17A in Psoriasis	1.38E-04	CCL20,CXCL1,CXCL3
Hematopoiesis from Multipotent Stem Cells	1.38E-04	CSF2,CSF3,IL7
Death Receptor Signaling	1.45E-04	BIRC3,NFKB2,NFKBIA,PARP12,PARP9,TNF
Antioxidant Action of Vitamin C	2.14E-04	CSF2,NFKB2,NFKBIA,PLA2G4E,STAT5A,TNF
Hepatic Fibrosis Signaling Pathway	2.88E-04	CCL2,IL1A,IRAK2,MMP13,MYLK3,NCF1,NFKB2,NFKBIA,RND1,TNF,WNT3A
IL-17 Signaling	5.89E-04	CCL2,CXCL1,CXCL10,CXCL11,IL6
IL-6 Signaling	7.08E-04	IL1A,IL6,NFKB2,NFKBIA,SOCS3,TNF
FAT10 Cancer Signaling Pathway	7.08E-04	IL6,NFKB2,NFKBIA,TNF
Communication between Innate and Adaptive Immune Cells	7.94E-04	CSF2,CXCL10,IL1A,IL6,TNF
Crosstalk between Dendritic Cells and Natural Killer Cells	8.71E-04	CSF2,IL6,LTB,NFKB2,TNF
Hepatic Fibrosis / Hepatic Stellate Cell Activation	9.12E-04	CCL2,CXCL3,IL1A,IL6,MMP13,NFKB2,TNF
Role of JAK family kinases in IL-6-type Cytokine Signaling	1.35E-03	IL6,SOCS3,STAT5A
Apoptosis Signaling	1.45E-03	BCL2A1,BIRC3,NFKB2,NFKBIA,TNF
PPAR Signaling	1.66E-03	IL1A,NFKB2,NFKBIA,STAT5A,TNF
Glucocorticoid Receptor Signaling	1.82E-03	CCL2,CSF2,CXCL3,IL6,NFKBIA,NPPA,POU2F2,STAT5A,TNF
Induction of Apoptosis by HIV1	1.95E-03	BIRC3,NFKB2,NFKBIA,TNF

## References

[1] Coleman CM, Frieman MB (2014). Coronaviruses: important emerging human pathogens. Journal of virology.

[2] Chen B, Tian E-K, He B, Tian L, Han R, Wang S (2020). Overview of lethal human coronaviruses. Signal Transduction and Targeted Therapy.

[3] Husnayain A, Shim E, Fuad A, Su EC (2020). Understanding the Community Risk Perceptions of the COVID-19 Outbreak in South Korea: Infodemiology Study. J Med Internet Res.

[4] Nguyen HC, Nguyen MH, Do BN, Tran CQ, Nguyen TTP, Pham KM (2020). People with Suspected COVID-19 Symptoms Were More Likely Depressed and Had Lower Health-Related Quality of Life: The Potential Benefit of Health Literacy. J Clin Med.

[5] Qin L, Sun Q, Wang Y, Wu KF, Chen M, Shia BC (2020). Prediction of Number of Cases of 2019 Novel Coronavirus (COVID-19) Using Social Media Search Index. Int J Environ Res Public Health.

[6] Liu SY, Kang XL, Wang CH, Chu H, Jen HJ, Lai HJ (2020). Protection procedures and preventions against the spread of coronavirus disease 2019 in healthcare settings for nursing personnel: Lessons from Taiwan. Aust Crit Care.

[7] Memish ZA, Perlman S, Van Kerkhove MD, Zumla A (2020). Middle East respiratory syndrome. Lancet.

[8] Blanco-Melo D, Nilsson-Payant BE, Liu WC, Uhl S, Hoagland D, Moller R (2020). Imbalanced Host Response to SARS-CoV-2 Drives Development of COVID-19. Cell.

[9] Harcourt J, Tamin A, Lu X, Kamili S, Sakthivel SK, Murray J (2020). Severe acute respiratory syndrome coronavirus 2 from patient with coronavirus disease, United States. Emerging infectious diseases.

[10] Yoshikawa T, Hill TE, Yoshikawa N, Popov VL, Galindo CL, Garner HR (2010). Dynamic innate immune responses of human bronchial epithelial cells to severe acute respiratory syndrome-associated coronavirus infection. PLoS One.

[11] Rayner RE, Makena P, Prasad GL, Cormet-Boyaka E (2019). Optimization of normal human bronchial epithelial (NHBE) cell 3D cultures for in vitro lung model studies. Scientific reports.

[12] Vishnubalaji R, Shaath H, Alajez NM (2020). Protein Coding and Long Noncoding RNA (lncRNA) Transcriptional Landscape in SARS-CoV-2 Infected Bronchial Epithelial Cells Highlight a Role for Interferon and Inflammatory Response. Genes.

[13] Lei C, Qian K, Li T, Zhang S, Fu W, Ding M (2020). Neutralization of SARS-CoV-2 spike pseudotyped virus by recombinant ACE2-Ig. Nat Commun.

[14] Ziegler CG, Allon SJ, Nyquist SK, Mbano IM, Miao VN, Tzouanas CN (2020). SARS-CoV-2 receptor ACE2 is an interferon-stimulated gene in human airway epithelial cells and is detected in specific cell subsets across tissues. Cell.

[15] Klahan S, Wong HS, Tu SH, Chou WH, Zhang YF, Ho TF (2017). Identification of genes and pathways related to lymphovascular invasion in breast cancer patients: A bioinformatics analysis of gene expression profiles. Tumour Biol.

[16] Huang da W, Sherman BT, Lempicki RA (2009). Systematic and integrative analysis of large gene lists using DAVID bioinformatics resources. Nat Protoc.

[17] Wong HS, Chang WC (2018). Losses of cytokines and chemokines are common genetic features of human cancers: the somatic copy number alterations are correlated with patient prognoses and therapeutic resistance. Oncoimmunology.

[18] Barrett T, Wilhite SE, Ledoux P, Evangelista C, Kim IF, Tomashevsky M (2013). NCBI GEO: archive for functional genomics data sets-update. Nucleic Acids Res.

[19] Durinck S, Spellman PT, Birney E, Huber W (2009). Mapping identifiers for the integration of genomic datasets with the R/Bioconductor package biomaRt. Nat Protoc.

[20] Zambon AC, Gaj S, Ho I, Hanspers K, Vranizan K, Evelo CT (2012). GO-Elite: a flexible solution for pathway and ontology over-representation. Bioinformatics.

[21] Gentleman RC, Carey VJ, Bates DM, Bolstad B, Dettling M, Dudoit S (2004). Bioconductor: open software development for computational biology and bioinformatics. Genome Biol.

[22] Sun Z, Wang C-Y, Lawson DA, Kwek S, Velozo HG, Owyong M (2018). Single-cell RNA sequencing reveals gene expression signatures of breast cancer-associated endothelial cells. Oncotarget.

[23] Cooke DL, McCoy DB, Halbach VV, Hetts SW, Amans MR, Dowd CF (2018). Endovascular Biopsy: In Vivo Cerebral Aneurysm Endothelial Cell Sampling and Gene Expression Analysis. Transl Stroke Res.

[24] Hagerling C, Gonzalez H, Salari K, Wang C-Y, Lin C, Robles I (2019). Immune effector monocyte-neutrophil cooperation induced by the primary tumor prevents metastatic progression of breast cancer. Proceedings of the National Academy of Sciences.

[25] Cheng L-C, Chao Y-J, Overman MJ, Wang C-Y, Phan NN, Chen Y-L (2020). Increased expression of secreted frizzled related protein 1 (SFRP1) predicts ampullary adenocarcinoma recurrence. Scientific reports.

[26] Phan NN, Liu S, Wang C-Y, Hsu H-P, Lai M-D, Li C-Y (2020). Overexpressed gene signature of EPH receptor A/B family in cancer patients-comprehensive analyses from the public high-throughput database. International Journal of Clinical and Experimental Pathology.

[27] Wang C-Y, Chang Y-C, Kuo Y-L, Lee K-T, Chen P-S, Cheung CHA (2019). Mutation of the PTCH1 gene predicts recurrence of breast cancer. Scientific reports.

[28] Wang C-Y, Chiao C-C, Phan NN, Li C-Y, Sun Z-D, Jiang J-Z (2020). Gene signatures and potential therapeutic targets of amino acid metabolism in estrogen receptor-positive breast cancer. American journal of cancer research.

[29] Wu P-S, Yen J-H, Wang C-Y, Chen P-Y, Hung J-H, Wu M-J (2020). 8-Hydroxydaidzein, an Isoflavone from Fermented Soybean, Induces Autophagy, Apoptosis, Differentiation, and Degradation of Oncoprotein BCR-ABL in K562 Cells. Biomedicines.

[30] Wang CY, Li CY, Hsu HP, Cho CY, Yen MC, Weng TY (2017). PSMB5 plays a dual role in cancer development and immunosuppression. Am J Cancer Res.

[31] Liu H-L, Yeh I-J, Phan NN, Wu Y-H, Yen M-C, Hung J-H (2020). Gene signatures of SARS-CoV/SARS-CoV-2-infected ferret lungs in short-and long-term models. Infection, Genetics and Evolution.

[32] Hsu HP, Wang CY, Hsieh PY, Fang JH, Chen YL (2020). Knockdown of serine/threonine-protein kinase 24 promotes tumorigenesis and myeloid-derived suppressor cell expansion in an orthotopic immunocompetent gastric cancer animal model. J Cancer.

[33] Ashburner M, Ball CA, Blake JA, Botstein D, Butler H, Cherry JM (2000). Gene ontology: tool for the unification of biology. The Gene Ontology Consortium. Nat Genet.

[34] Subramanian A, Tamayo P, Mootha VK, Mukherjee S, Ebert BL, Gillette MA (2005). Gene set enrichment analysis: a knowledge-based approach for interpreting genome-wide expression profiles. Proc Natl Acad Sci U S A.

[35] Madjid M, Safavi-Naeini P, Solomon SD, Vardeny O (2020). Potential effects of coronaviruses on the cardiovascular system: a review. JAMA cardiology.

[36] Market M, Angka L, Martel AB, Bastin D, Olanubi O, Tennakoon G (2020). Flattening the COVID-19 Curve With Natural Killer Cell Based Immunotherapies. Frontiers in Immunology.

[37] Mehta P, McAuley DF, Brown M, Sanchez E, Tattersall RS, Manson JJ (2020). COVID-19: consider cytokine storm syndromes and immunosuppression. Lancet.

[38] Di Gennaro F, Pizzol D, Marotta C, Antunes M, Racalbuto V, Veronese N Coronavirus Diseases (COVID-19) Current Status and Future Perspectives: A Narrative Review. 2020; 17: 2690.

[39] Baas T, Taubenberger JK, Chong PY, Chui P, Katze MG (2006). SARS-CoV virus-host interactions and comparative etiologies of acute respiratory distress syndrome as determined by transcriptional and cytokine profiling of formalin-fixed paraffin-embedded tissues. Journal of interferon & cytokine research.

[40] Pedersen SF, Ho Y-C (2020). SARS-CoV-2: a storm is raging. The Journal of clinical investigation.

[41] Chen W (2020). A potential treatment of COVID-19 with TGF-β blockade. International Journal of Biological Sciences.

[42] Wang CY, Lu CY, Li SW, Lai CC, Hua CH, Huang SH (2017). SARS coronavirus papain-like protease up-regulates the collagen expression through non-Samd TGF-beta1 signaling. Virus Res.

[43] Baas T, Taubenberger JK, Chong PY, Chui P, Katze MG (2006). SARS-CoV virus-host interactions and comparative etiologies of acute respiratory distress syndrome as determined by transcriptional and cytokine profiling of formalin-fixed paraffin-embedded tissues. J Interferon Cytokine Res.

[44] Irham LM, Chou WH, Calkins MJ, Adikusuma W, Hsieh SL, Chang WC (2020). Genetic variants that influence SARS-CoV-2 receptor TMPRSS2 expression among population cohorts from multiple continents. Biochem Biophys Res Commun.

[45] Hoffmann M, Kleine-Weber H, Schroeder S, Kruger N, Herrler T, Erichsen S (2020). SARS-CoV-2 Cell Entry Depends on ACE2 and TMPRSS2 and Is Blocked by a Clinically Proven Protease Inhibitor. Cell.

[46] Bosch-Barrera J, Martin-Castillo B, Buxó M, Brunet J, Encinar JA, Menendez JA (2020). Silibinin and SARS-CoV-2: Dual Targeting of Host Cytokine Storm and Virus Replication Machinery for Clinical Management of COVID-19 Patients. Journal of Clinical Medicine.

[47] Clementi N, Ferrarese R, Criscuolo E, Diotti RA, Castelli M, Scagnolari C Interferon-β-1a Inhibition of Severe Acute Respiratory Syndrome-Coronavirus 2 In Vitro When Administered After Virus Infection. The Journal of Infectious Diseases.

[48] Chen Z, Song X, Li Q, Xie L, Guo T, Su T (2019). Androgen receptor-activated enhancers simultaneously regulate oncogene TMPRSS2 and lncRNA PRCAT38 in prostate cancer. Cells.

[49] Clinckemalie L, Spans L, Dubois V, Laurent M, Helsen C, Joniau S (2013). Androgen regulation of the TMPRSS2 gene and the effect of a SNP in an androgen response element. Molecular endocrinology.

[50] Matsuyama S, Nao N, Shirato K, Kawase M, Saito S, Takayama I (2020). Enhanced isolation of SARS-CoV-2 by TMPRSS2-expressing cells. Proc Natl Acad Sci U S A.

[51] Bacellar O, Faria D, Nascimento M, Cardoso TM, Gollob KJ, Dutra WO (2009). Interleukin 17 production among patients with American cutaneous leishmaniasis. The Journal of infectious diseases.

[52] Malavolta M, Giacconi R, Brunetti D, Provinciali M, Maggi F (2020). Exploring the Relevance of Senotherapeutics for the Current SARS-CoV-2 Emergency and Similar Future Global Health Threats. Cells.

[53] Gao ZW, Wang X, Lin F, Dong K The correlation between SARS-CoV-2 infection and rheumatic disease. Autoimmun Rev. 2020: 102557.

[54] Liu C, Chen Z, Hu Y, Ji H, Yu D, Shen W (2018). Complemented Palindromic Small RNAs First Discovered from SARS Coronavirus. Genes (Basel).

[55] Risitano AM, Mastellos DC, Huber-Lang M, Yancopoulou D, Garlanda C, Ciceri F (2020). Complement as a target in COVID-19?. Nat Rev Immunol.

